# Integrating Survivorship Care Into a Radiation Medicine Program

**DOI:** 10.7759/cureus.8013

**Published:** 2020-05-07

**Authors:** Shaziya Malam, Belinda Lawrence, Cari Bradley, Kimberly M McBride, Ashley Clement, Tatiana Conrad, M.Cheryl Noronha, Jeanette K Wong, Rachel A Woo, Zahra Kassam

**Affiliations:** 1 Radiation Oncology, Southlake Regional Health Centre, Newmarket, CAN

**Keywords:** cancer, survivorship, radiation therapy, education

## Abstract

Introduction

An important but often overlooked component of caring for cancer patients is survivorship care, provided after the completion of active treatment in order to facilitate transition into the next surveillance phase. A survivorship program was developed to deliver a one-on-one education session on healthy lifestyle behaviours and available resources to help patients transition to their post-treatment life. This study reports the outcome of this pilot survivorship care program provided to breast cancer patients completing radiation therapy. Program delivery format and content were evaluated for effectiveness, applicability, and feasibility.

Methods and materials

Between March 2017 and August 2018, 124 breast cancer patients, nearing completion of their curative intent radiation treatments, participated in this centre-specific survivorship program. The survivorship program entailed a one on one education session delivered to breast cancer patients within the last two weeks of their radiation treatment. Participants were provided a Microsoft PowerPoint presentation, information pamphlet, and evaluation form to provide feedback on materials and presentation. Survivorship education sessions were delivered by study staff or staff scheduled in the Pre-Radiotherapy Patient Assessment role. Follow-up phone calls were conducted post-session delivery to determine the ongoing applicability of survivorship material. Staff was also given an evaluation form upon completion of the trial to measure the session feasibility.

Results

Of the 124 participants in the study, 69 (56%) provided feedback. Results showed that 98% of participants felt the information provided either confirmed what they were already doing (44%) or encouraged them to consider a lifestyle change (54%). Additionally, 70% reported feeling more confident after completing the session. Staff survey results reported that 87.5% agreed or strongly agreed that these sessions were beneficial and valuable to patients

Conclusions

Delivering one-on-one education sessions to individual participants focusing on healthy lifestyle measures garnered a positive response from participants, increasing their confidence and knowledge for making lifestyle changes. While staff survey results pointed strongly in favour of continuing with the survivorship sessions, it was shown that the methods of delivery trialed in this study were not feasible to be implemented on a larger scale. With some workflow modification, implementing a survivorship care program in our cancer centre is a possible and important aspect of a patient’s treatment journey.

## Introduction

There are numerous aspects to providing care to individuals with breast cancer, from delivering active treatment to offering social support. A significant but often overlooked component of caring for breast cancer patients is survivorship care, provided after the completion of active treatment in order to facilitate transition into the next surveillance phase [1]. Cancer’s burden, which includes physical and psychosocial issues, does not end when active treatment is completed. Some long-term issues patients may face include ongoing pain, fatigue, lymphedema, heart disease, difficulty sleeping, weight gain, sexual dysfunction, stress, and financial issues [2]. Providing survivorship care, particularly education about healthy lifestyle behaviours, allows for the individualization of the treatment delivery model to the specific needs of individual patients while affording patients the resources needed to successfully transition to life post-treatment.

More and more people are living longer after a cancer diagnosis and treatment. The Canadian Cancer Society reported that at the beginning of 2009, there were more than 800 000 Canadians who had had a cancer diagnosis in the last 10 years [3]. Breast cancer makes up about 25% of all new cancer cases amongst Canadian women and about 87% of breast cancer patients are expected to live at least five years post diagnosis [4]. One in 8 Canadian women is expected to be diagnosed with breast cancer in their life [5]. As the prevalence of cancer grows in Canada, so does the need to have a comprehensive survivorship strategy.

There is overwhelming evidence that the current state of survivorship care is falling short to meet the needs of patients. In 2018, the Canadian Partnership Against Cancer (CPAC) published a report titled Living With Cancer- a Report on the Patient Experience [6]. Presenting patients’ experiences with their cancer journey, this report highlights many gaps in survivorship care across Canada. According to CPAC, about 67.7% of patients face physical, emotional and practical challenges after treatment completion [6]. Most cancer survivors need someone to talk to about concerns, and more information about how to address them [6]. Often, patients are not able to get help and if they do, 34% of them wait more than three months to receive it [6]. Many times, information received is not useful [6]. Thus, it is evident that more comprehensive care is required for cancer survivors and we must do better to serve Canadians. 

In 2006, the Institute of Medicine recommended that every cancer patient receive an individualized survivorship care plan that includes guidelines for monitoring and maintaining their health [1]. Radiation therapy is well-positioned to offer survivorship education to breast cancer patients since it is often the last step in active treatment before patients are transitioned into the survivorship phase of their cancer journey [7]. Additionally, radiation therapists see breast cancer patients everyday for 3-5 weeks and are able to build good rapport based on trust and understanding. At our centre, a patient follow-up call is completed one-week post-radiation treatment, and survivorship care has been anecdotally identified as a gap in care when gathering feedback. Currently, there is no individual survivorship education provided to breast cancer patients when completing radiation therapy treatment at our centre. This study reports the outcomes of a pilot survivorship care program, designed to deliver a one on one education session on healthy lifestyle behaviours and available resources to help patients transition to their post-treatment life, provided to breast cancer patients completing radiation therapy. Program delivery format and content was evaluated for effectiveness, applicability and feasibility. 

## Materials and methods

Program and material development

Considerations given to the survivorship program model included ease of implementation, integration into current department workflow, and delivery of education session. Survivorship sessions were scheduled near the completion of radiation treatment courses since this often marks the end of active treatment for breast cancer. Survivorship education was also provided immediately after radiation therapy appointments to prevent extra visits for patients.

The survivorship program was developed utilizing multi-disciplinary collaboration. Staff dieticians, social workers, a nurse practitioner and two Radiation Oncologists were consulted regarding survivorship topics, materials, and content. Survivorship topics and content was also based on recommendations and findings from other published sources [1-2,8-14]. Additionally, the material was generalized to be applicable to all breast cancer stages.

Materials developed for the study included a Microsoft PowerPoint presentation and an information pamphlet (Appendix A). Material content covered exercise, nutrition, stress relief, smoking cessation, changes to sexuality, available community resources, patient programs offered at our centre, and disease surveillance post-treatment. Prior to implementation, the information pamphlet was reviewed by two current breast cancer patients, the Regional Lead for Person-Centred Care and two Radiation Oncologists.

Implementation

Between March 2017 and August 2018, a convenience sample of 124 female breast cancer patients, treated in the curative setting, nearing completion of their radiation treatments agreed to participate in this centre-specific survivorship care program. Inclusion criteria were female breast cancer patients stages 0-III, within two weeks of the end of radiation treatment. No exclusion criteria were utilized. 

The survivorship program entailed a one-on-one education session given to breast cancer patients within the last two weeks of their radiation treatment. Sessions were approximately 20 minutes in length and were provided by eight participating radiation therapists, each of whom was trained to deliver the sessions. During each session, patients were presented with a Microsoft PowerPoint presentation and provided a study-specific education pamphlet that summarized the material reviewed. After each session, participants were given an opportunity to ask any questions. Survivorship education sessions were delivered using two workflows, conducted by either study staff or staff scheduled in our Pre-Radiotherapy Patient Assessment role, which has been previously reported (Unpublished abstract: Smith S, Culhane J, Woo R. Implementation of a Pre-radiation Therapy Patient Assessment Role at the Stronach Regional Cancer Centre (SRCC). 10th Annual Radiation Therapy Conference; June 2014).

At the end of the education sessions, patients were given a study-specific evaluation form to complete and submit to study staff. This form was developed based on a study published by Trask et al. and included questions to evaluate the session content, delivery method, and participant experience [15]. Participants were asked to rate how well the session met the objectives stated in the beginning, how comfortable they felt incorporating what they learned in their everyday lives and if they learned any helpful tips with regards to healthy living. Participants were also asked to evaluate the strengths/weaknesses of the survivorship session and provide feedback for session improvement. Questions were a mix of close-ended questions with a Likert scale for agreement/disagreement (1= Strongly Disagree, 5= Strongly Agree), as well as a number of open-ended questions. See Appendix B for patient evaluation form. 

In addition, patients received a follow-up phone call 4-12 weeks post-session delivery to evaluate the ongoing applicability of the survivorship education materials. If the participant was not able to be reached, two attempts were made, two to four weeks apart. Patients were asked several pre-defined open-ended questions, including whether they had made any changes to their lifestyle as a result of the survivorship session, the most useful parts of the session, and the effectiveness of the session delivery method. 

Finally, the staff involved in the delivery of the education sessions was given a study-specific evaluation form to complete. This evaluation assessed staff experience in regards to the session, how well the session was received by patients, the workload associated with conducting the sessions, and the feasibility of incorporating a survivorship education session into daily department workflow. Evaluation design was similar to the patient evaluation form, consisting of close-ended questions with a Likert scale for agreement/disagreement (1 = Strongly Disagree, 5 = Strongly Agree) and several open-ended questions (Appendix C). 

After a discussion with the local Research Ethics Board (REB) this project was deemed to be a quality improvement initiative to identify and meet an existing gap in care and an exemption from REB approval was provided. All participants involved in the research gave verbal consent to participate. Study involvement was voluntary, and participants were informed of the option to withdraw consent at any time.

## Results

Overall, 124 patients, aged 32-87 with stage 0 to III breast cancer, participated in the study. There was a response rate of 56% for the participant evaluation forms administered at the time of session delivery (69/124). Four evaluation forms were incomplete when returned; however the completed responses were still included in results. In addition, 56 follow up phones calls were completed 4-12 weeks after the completion of treatment (45% response rate). One hundred percent of staff (8/8) completed staff evaluation forms at study completion. 

Participant evaluation

**Figure 1:**
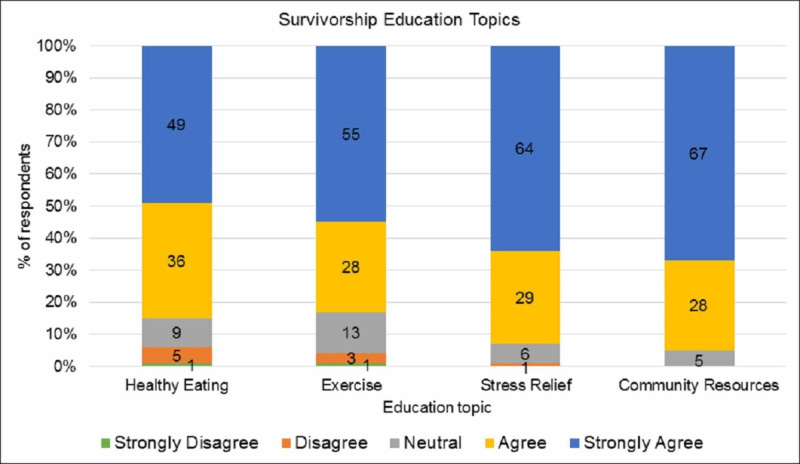
Survivorship education topics (n = 69) Study participants were asked if they learned helpful tips about each of these topics: healthy eating, exercise, stress relief, and community resources.

**Figure 2:**
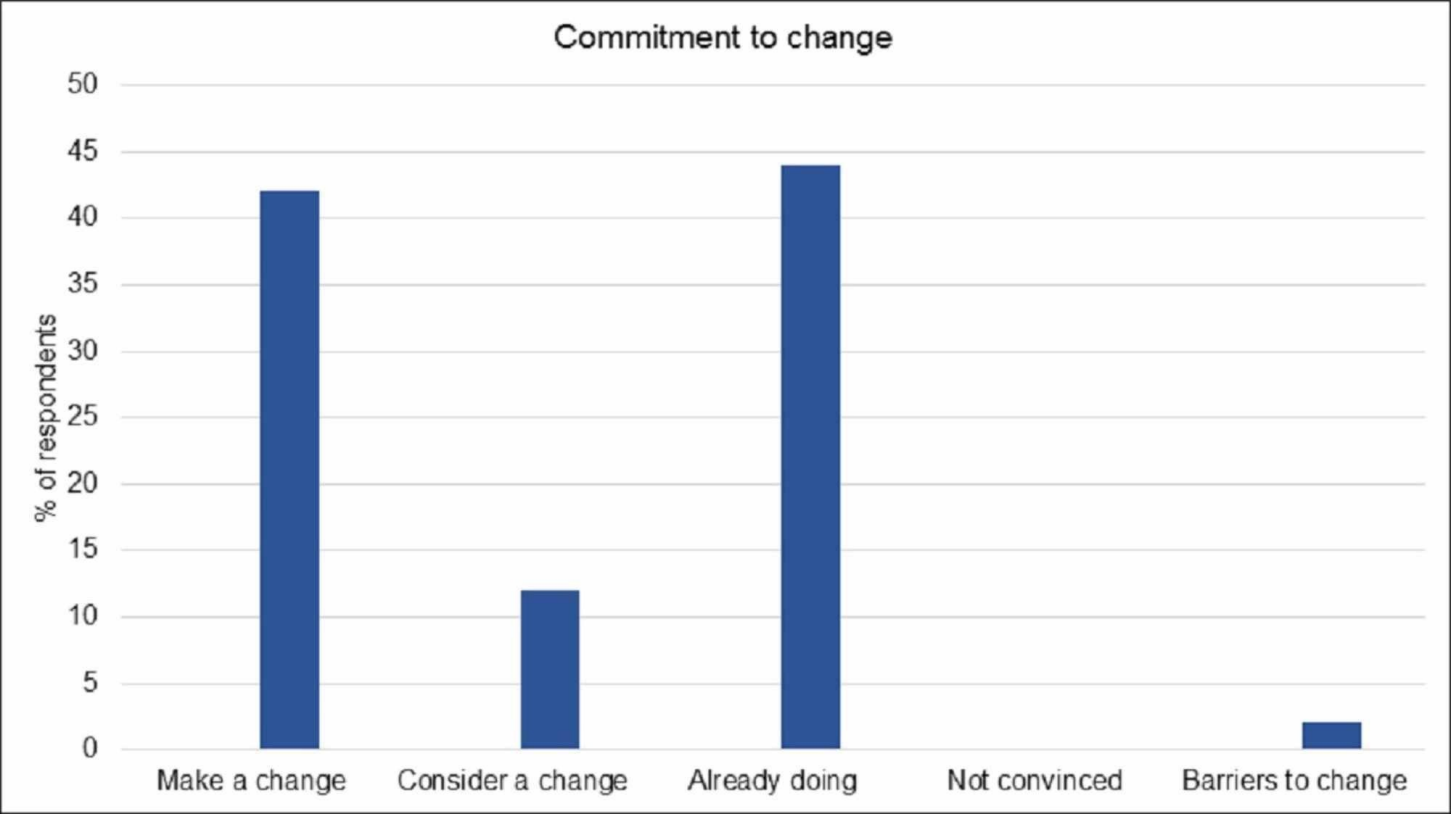
Commitment to change (n = 69) Participants were asked to rate their commitment to making lifestyle changes after receiving the survivorship education.

Patients were asked if they learned helpful tips about each topic discussed during the survivorship session and to rate each section using a 5-step Likert scale. See Figure 1 for results. Overall, results show that participants learned helpful tips for making lifestyle changes, namely healthy eating, exercise, stress management, and community resources that can be accessed. Eighty-five percent of participants agreed/strongly agreed that they learned helpful tips regarding healthy eating, 83% for exercise, 93% for stress relief, and 95% for community resources. In addition, 98% of participants surveyed felt the information provided either confirmed what they were already doing (44%) or prompted them to make or consider a change to their lifestyle (54%) (Figure 2). Participants were also asked if they faced any barriers to making lifestyle changes. Many stated that there were no barriers and barriers that were identified include stubbornness, old habits, willingness to change, and lack of time. Eighty-four percent of participants agreed/strongly agreed that the information provided was applicable to them and 82% agreed/strongly agreed that the learning method used was effective. Furthermore, 70% reported feeling more confident in themselves since completing the educational activity.

With regards to the open-ended questions, comments made regarding the survivorship program include “it was an excellent transition,”, and “knowing support is available.” Participants commented that the most useful part of the survivorship sessions was “learning how to improve my health and life” and the comfort of knowing that “support programs, people, etc. are available to me.” One participant wrote that it was helpful “knowing that what I already was doing was my best for my health.” They appreciated that the survivorship sessions brought them “ease of mind with what to do after” and “the sense that you are still being thought of and cared for.” Another commented that “the presentation and booklet are very helpful and very encouraging. Thank you for the emotional support in addition to the treatments.” Many patients noted that the survivorship sessions confirmed what they were already doing. “The meeting was useful/ helpful. I used it as a reminder/reinforcement of how I already lead my life.” 

Follow-up calls

**Figure 3:**
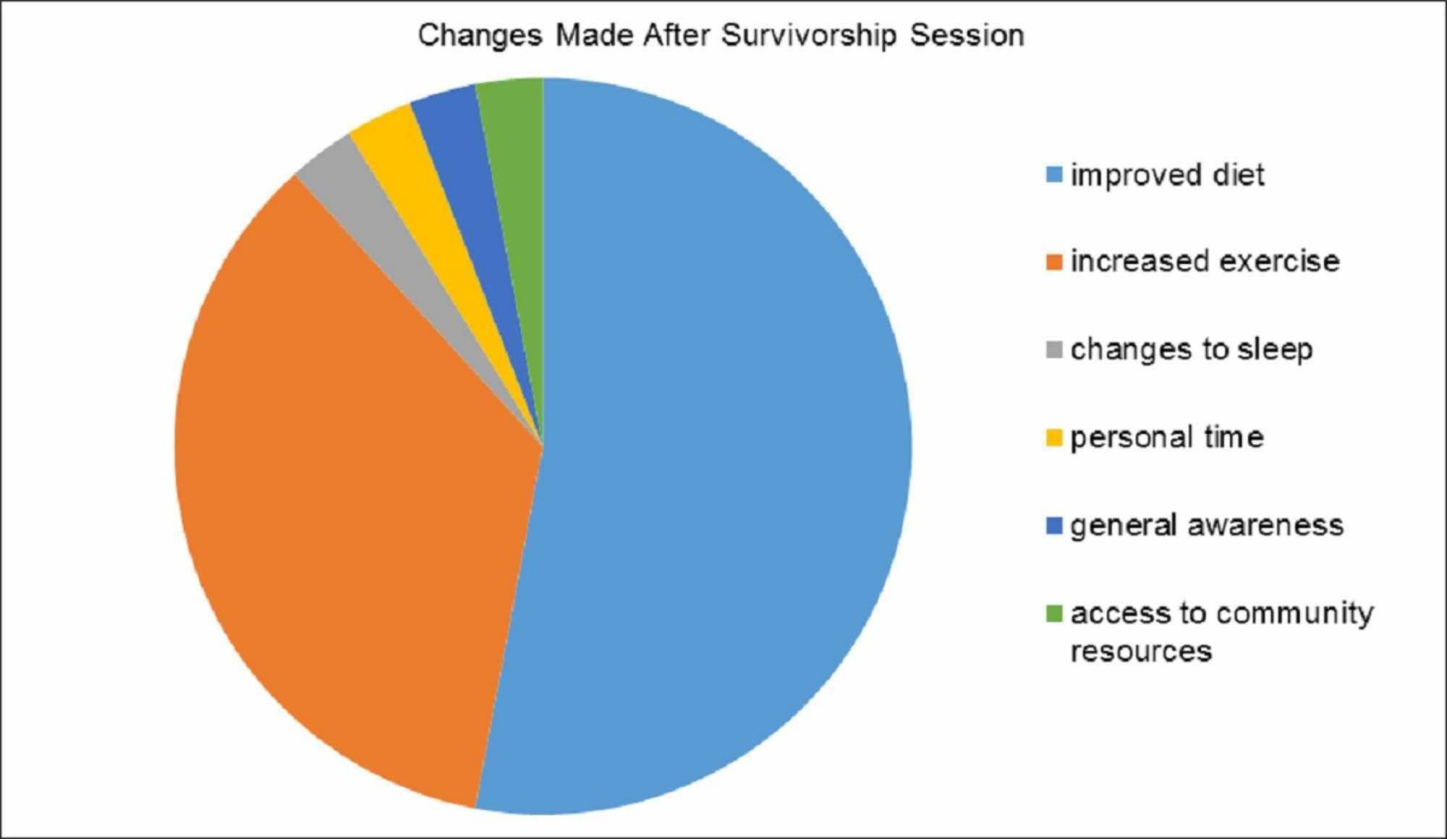
Patient-reported lifestyle changes made after the completion of survivorship sessions (n = 56)

Participants who completed the survivorship sessions received a follow-up phone call 4-12 weeks after completion of treatment to determine the ongoing applicability of the survivorship sessions. At the end of the phone call, participants were given an opportunity to bring forth any pending questions/concerns. Out of the 124 patients involved in the study, 56 received follow-up calls. Several patients were unavailable or unreachable when calls were placed. Overall, 26 of the 56 patients (46%) had made lifestyle changes since completing the sessions. Most changes made included an improved diet and increased exercise (Figure 3).

**Figure 4:**
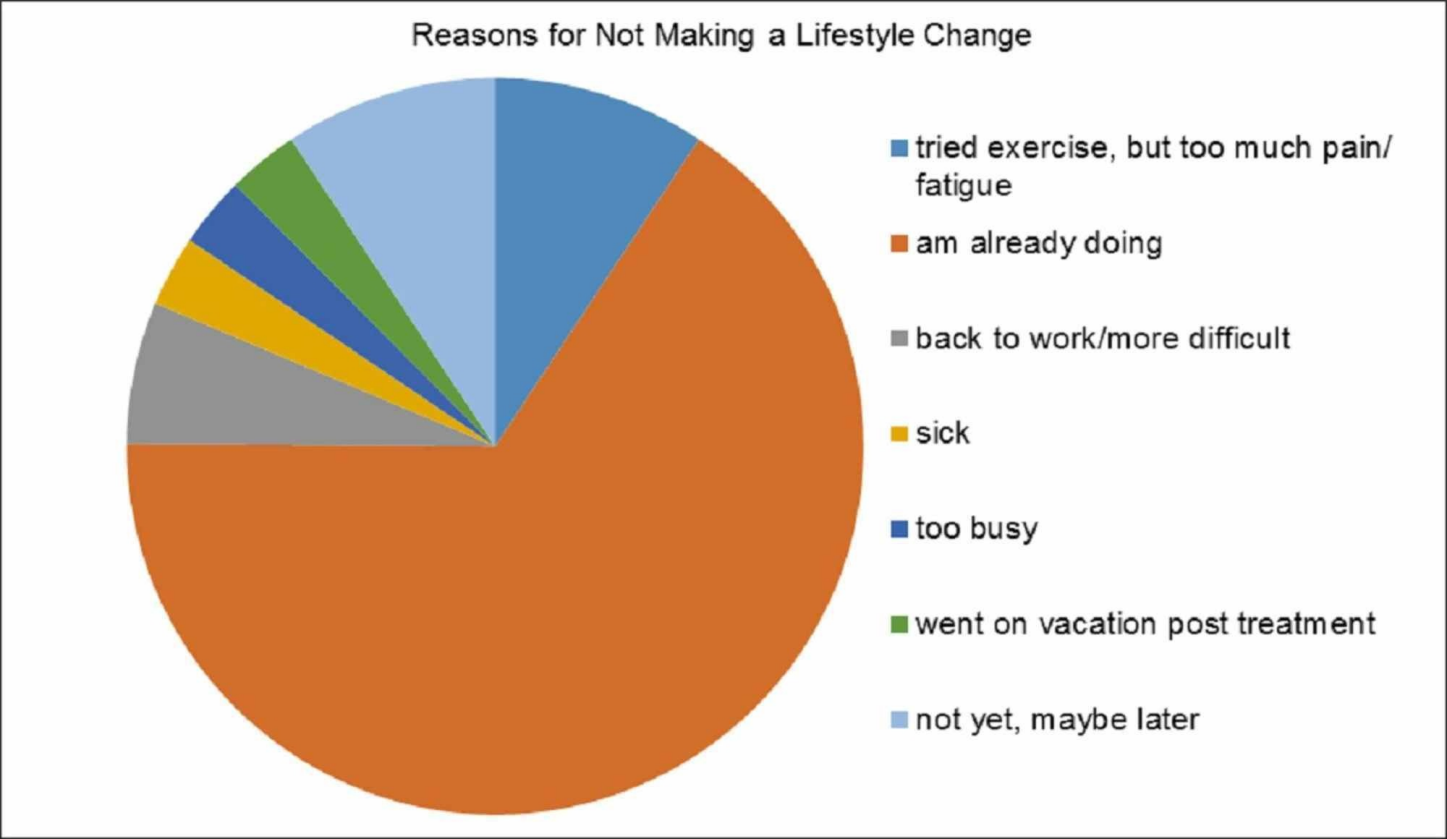
Patient-reported reasons for not making any lifestyle changes after attending the survivorship education session (n = 56)

Of those 30 patients (54%) who had not made changes, 21 patients stated it was because they were already doing what was recommended. Other reasons for not making any changes included patient-specific factors, such as too much pain or illness, being busy, or difficulties after returning to work. Three patients reported that although they had not made any changes yet, they plan to make them in the future. See Figure 4 for the results. 

In addition, patients were asked whether the format of the sessions was appropriate. Of the 56 participants called, 54% preferred one-on-one sessions to group sessions, 23% stated either one-on-one or group sessions would be beneficial, and 18% stated they would have preferred group sessions. Finally, 98% of patients stated that these sessions would be beneficial to others, with 2% stating they were not sure.

Staff evaluation

Of the eight staff involved in survivorship session delivery, 100% returned a staff evaluation form at study completion. Of the evaluations provided by the eight participating staff, seven (87.5%) agreed/strongly agreed that the sessions were useful to patients, and seven (87.5%) found value in continuing the education sessions. In addition, five (62.5%) staff felt that the sessions were able to be completed in a timely manner.

## Discussion

This study focused on one Regional Cancer Centre’s experience when incorporating survivorship care into the breast cancer patient population. These sessions provided patients with an opportunity to learn about how to better care for themselves going forward, ask any questions they may have, and feel more in control of their new normal post-treatment. 

Results from this study pointed out in favour of incorporating survivorship care into the standard of practice for breast cancer patients receiving radiation treatment. Participants responded well to the information provided and generally felt more confident in their abilities to transition to a healthful life post treatment as a result of these sessions. Being diagnosed and treated for cancer can leave one feeling hopeless and at a loss of control over one’s own life [16]. These sessions allowed patients to regain some control over their situation and provided them with the tools they needed to move forward in their cancer journey. 

Another study objective was to identify whether the delivery format utilized was most effective for patient learning. It has been previously reported that one-on-one patient education is effective in improving the quality of life for patients with heart failure [17]. Group education sessions have shown to have low attendance due to factors such as additional travel to the clinic, convenience, work conflict, or anxiety due to disease, treatment, or other factors [18]. In this study, most participants stated that the delivery method utilized was an effective method for this type of information and that it is the delivery format of choice. Many participants expressed that this format is more personable and gave them the opportunity to ask questions they may not have been comfortable asking during a group session. Due to the “three methods of learning for each session- see, listen, read”, participants noted that this format compelled them to be attentive and ensure understanding of the content.

In addition, the results of this study show that survivorship education helped increase patient confidence in themselves. At the time of session delivery, nearly all participants identified that they would consider making lifestyle changes if they were not already following our recommendations. At the time of follow- up calls one to three months after the completion of treatment, approximately half of participants had made changes to their lifestyle. The majority of those who had not made changes were already practicing what was recommended. These results are in line with statistics reported in the literature, namely that between 30% and 60% of patients are expected to make lifestyle changes after cancer diagnosis [16,19]. 

Incorporating community resources into survivorship resources provides patients an opportunity to utilize support programs available to them outside of the cancer centre. Chan et al. highlight the importance of providing this opportunity considering many cancer patients try to avoid triggering negative memories of their past cancer experience, and returning to the cancer centre can be one of them [20].

Interestingly, when asked if there were any barriers to making lifestyle changes, many participants stated that there were no barriers. Barriers that were identified include stubbornness, old habits, willingness to change and lack of time. At the start of the study, it was expected that socio-economic barriers, such as access to healthy food or affordability of healthy food options/ exercise programs would play a bigger role than was actually identified from study results, as these have been previously cited as challenging [21,22].

While staff survey results pointed strongly in favour of continuing with the survivorship sessions, it was shown that the two methods of delivery trialed in this study were not feasible to be implemented on a larger scale. Staff found that it was difficult incorporating these sessions into very busy, unpredictable schedules and coordinating all of the survivorship sessions. Unstructured timing and scheduling of the sessions was one of the challenges faced by staff when striving to complete them. In addition, a lack of staff, space and time resources available proved to be a barrier for the trialed workflows. More detailed results on the staff evaluation are discussed in an accompanying paper (Unpublished data: Malam S, Lawrence B, Bradley C, et al. The Challenges with Implementing Survivorship Care into a Radiation Medicine Program: One Cancer Centre’s Experience; 2019).

Study limitations

There were a number of limitations to this study. First, the pilot included only one format for education delivery. The feasibility and response to group sessions were not tested. Participant preference for delivery format was partly based on perceptions of what they thought a group session would be like. In addition, not all participants returned an evaluation form at session completion. Moreover, due to time and staffing constraints, multiple attempts to reach participants over the phone were not viable in this study. Thus, we cannot know if the results would have been altered if all participants in the study had provided feedback, whether written or over the phone. The responses were also kept anonymous; therefore we cannot be certain that 45% of participants who were reached in the follow-up phone calls were not fully encompassed in the 56% of participating survey respondents. Furthermore, any lifestyle changes made post-treatment may not be directly due to the survivorship program and could be due to a multitude of other contributing factors. Finally, there were some logistical limitations that may have affected the effectiveness of the study, such as language barriers and scheduling conflicts among some participants. 

Future directions

Future directions for this study include implementing the survivorship program into the department. The plan would be to initially target breast cancer patients completing radiation treatment, with the plan to eventually roll out to all patients receiving dose radiation therapy with a curative intent. The information pamphlet and presentation will require some revision, as current content is tailored specifically for breast cancer patients. More general follow-up information will need to be adopted for other cancer sites. Additionally, appropriate workflow will need to be identified in order to implement this program department wide. This includes workflow processes for appointment scheduling, location, and staff scheduling in order to accommodate all of the survivorship sessions. This initiative will also help the cancer centre collaborate with community programs to help create a seamless transition of care back to the community. A follow-up paper outlining further details of program implementation feasibility, challenges, and issues are currently in development as well.

## Conclusions

Post-treatment survivorship care is an integral component of cancer care for patients undergoing radiation therapy. This study evaluated the applicability, effectiveness and feasibility of implementing a simple survivorship program into one Regional Cancer Centre. Delivering one-on-one education sessions to individual participants focusing on healthy lifestyle measures, garnered a positive response from participants, increasing their confidence and knowledge for making lifestyle improvements. Survivorship care has the ability to improve quality of life for patients post-treatment, which can lend itself to improved outcomes and overall survival. It enables patients to feel empowered and feel more in control of their lives. Having the ability to provide these sessions to patients completing their radiation therapy treatments proves to be an important aspect of a patient’s treatment journey and allows them to embrace their new normal after cancer. 

## Appendices

Appendix A

Life After Treatment- Information Pamphlet

What now?

Congratulations!

You are completing your treatments!

As relieved and excited as you may be about finishing your treatments, you may also be feeling worried and scared. Unexpected anxiety and uncertainty about the future is very common and perfectly normal when you finish treatment. You are not alone.

The end of cancer treatment is often a time of transition. One of the most challenging things after treatment is not knowing what happens next. It is also about finding your new normal.  

To help you with the next steps, we are providing you with information about life after treatment. We explain how to care for yourself now that you are finished.

It is important to identify your needs and to think about the support you might need as a cancer survivor. Whatever you identify your needs to be, give yourself time to adapt. Take it ONE DAY AT A TIME.

GOOD HABITS for HEALTH

Nutrition

The fuel your body uses to fight disease is critical to your overall wellbeing. Choose your food wisely. 

Every bite matters!

The balance of evidence also suggests that a whole food, plant-based diet may help prevent, treat, slow, and even reverse cancer progression. See Table 1 below for nutrition guidelines. 

**Table 1:** Nutrition Guidelines

Nutrition Guidelines
Focus on eating mostly plant foods in their whole unprocessed form (i.e vegetables, nuts, seeds, beans, sprouts, lentils, and fruit)
Limit or avoid red meat, processed meats, trans fats (ex. Doughnuts, fried fast food, cookies, pies, margarine)
Limit or avoid charred and high-salted foods
Eat healthy fats (i.e avocados, nuts, seeds, extra virgin olive oil) and AVOID flours and sugars
Consider fruits, legumes, whole grains, vegetables as carbohydrate sources
Drink 6-8 glasses of water per day (avoid juice and sodas)
Read labels and try to purchase foods with ingredients you know
Try to avoid foods with artificial colours/flavours/sweeteners (i.e aspartame, Splenda) and additives

Exercise

Existing data strongly suggests that exercise is safe and doable during and after cancer treatment. It can also improve physical functioning, fatigue, and many aspects of quality of life. Many studies have shown that physically active cancer survivors have a lower risk of cancer recurrences and improved survival compared with those who are not active.

Also, in cancer survivors, exercise has been shown to improve

·          heart health

·          muscle strength

·          body composition

·          fatigue

·          anxiety

·          depression

·          self-esteem

·          happiness

·          quality of life

The goal is to find a way to add some kind of movement into your daily routine. Some movement is much better than no movement at all.

Examples of exercise can include walking, exercise bike, treadmill, household chores, yoga, and tai chi. See Table 2 below for some exercise guidelines.

**Table 2:** Exercise Guidelines

Exercise Guidelines
Start SLOW
Try exercising during the time of day when you feel as though you have the most energy
Try to do some form of exercise every day
Vary activities to include strength, flexibility, and aerobic activities
Drink plenty of fluids prior to, during and after workouts

Tips to stay motivated:

•      try to establish a routine

•      find someone who can participate with you

•      write out a plan of action

•      journal your activities and progress or make a chart

Talk with your doctor prior to starting any workout routine.

Stress relief

While some may think that chronic stress is just “part of life”, there’s strong evidence that shows we should take it more seriously. Research shows that long periods of high stress is not good for your health. It increases our chances of getting colds, slows wound healing, decreases immune function, and raises blood pressure and cholesterol levels.

It is important to increase our stress awareness by paying attention to our symptoms. These can include depression, stomach and/or chest pains, rapid heartbeat, and insomnia. Recognizing your stress levels can help you be proactive towards finding ways to lower them. Some helpful ways to manage stress are listed in Table 3 below. 

**Table 3:** Helpful Ways to Manage Stress

Helpful Ways to Manage Stress
Get regular follow-up screening
Exercise regularly
Avoid smoking/tobacco
Eat a healthy diet
Have a good network of family and friends. Talk to those in your support system of your concerns.
Meditation and/or prayer
Massage
Nature walks
Pets
Hobbies, music, reading
Distractions like comedy movies

The thought of cancer recurring may sometimes feel completely out of our control but all situations have controllable and uncontrollable aspects. It’s true that to some extent, you have no control over whether or not your cancer recurs, but you can have some control over the situation by managing your stress.

Gradually, this effort will help lower your stress levels. Most cancer survivors also report that the fear of recurrence fades with time.

Smoking cessation

Tobacco smoke negatively hurts every part of the body. Even if someone else in your life smokes and not you, the smoke still harms your body. If you do smoke, quitting is one of the best things you can do to for your health. Even reducing the number of cigarettes you have each day is a great start. Although you are finished your radiation treatments, it is never too late to quit. By quitting, you can lower your risk of things like getting a new cancer or having your cancer come back.

If you are thinking of quitting, there are many programs and resources available to help. Ask any of your health care practitioners for more information. For example, we can send a letter to your family doctor or pharmacist to let them know that you would like more support to quit. We can also give you a list of pharmacies in your area that offer quit counselling and support. If you are finished treatment and would still like support, you can always talk to your family doctor or your local pharmacist. The Canadian Cancer Society also offers a comprehensive counseling service to help you quit.

Canadian Cancer Society Smokers’ HelpLine

Phone: 1-877-513-5333

Website: smokershelpline.ca

Changes in sexuality

Cancer and its treatment may affect your sexual function and feelings. If you and/or your partner have any questions or concerns, please speak to your Radiation Oncologist or Oncology Nurse.

Please visit the Patient and Family Resource Centre in the lobby of the Stronach Regional Cancer Centre for additional information and resources related to your treatment.

Please refer to the Canadian Cancer Society Book: Cancer and Sexuality.

A good online resource is Interventions to Address Sexual Problems in People with Cancer guideline at www.cancercare.on.ca/psoguidelines

If you would like to read more guides on how to cope with side effects please refer to www.cancercare.on.ca/symptoms

Support groups

Many cancer survivors find it very useful to attend support or wellness groups in their local communities after finishing cancer treatments. Benefits can include reduced stress, improved recovery and an overall better sense of wellbeing. There are a number of support groups available to you, both at the SRCC as well as in your own communities. Below, you will find a few of the many resources that you can take advantage of.

Stronach Regional Cancer Centre

The Stronach Regional Cancer Centre runs a free cancer survivorship program called Pathways to Wellness. The program consists of six two hour weekly sessions designed to provide education and strategies to manage stress and anxiety, improve nutrition, maximize physical activity and manage health concerns after a cancer diagnosis. For more information and to register contact Debbie Walsh 905 895-4521 ext. 6627.

Also, the Patient Resource Library on the Main Level in the Lobby can be very useful in providing you with useful information. Here you will find a wide variety of educational materials which may be of great help to you.

Canadian Cancer Society

The Canadian Cancer Society has a lot of information regarding local support groups/ programs throughout the Greater Toronto Area. You can find local support groups/ programs by typing in your postal code here: http://info.cancer.ca/CSD/searchCon.aspx?id=3172&Lang=E&sri=N.

Doane House Hospice

Doane House Hospice is located in Newmarket and runs a number of wellness groups for cancer survivors, such as art therapy, caregiver support, and mind-body connection.

Phone: 905-967-0259

Website: http://www.doanehospice.org/

Email:

Hospice King-Aurora

Hospice King-Aurora is located in Aurora and serves the Aurora, Richmond Hill and Markham areas. Free programs available include yoga, meditation and Picking up the Pieces specifically for cancer survivors.

Phone: 905-727- 6815

Website: http://www.hospicekingaurora.com/

Email:

Disease surveillance

Surveillance after Breast Cancer

You will see your Radiation Oncologist for a Follow-up visit 4-6 weeks after finishing your radiation treatment. After this, you will be followed by members of your Health Care Team. Typically, this can be a family doctor, medical oncologist, or other medical practitioner.

Appendix B

Survivorship Post-Radiation Therapy: Patient Evaluation Form

Please help us to improve this survivorship session by completing this evaluation form. Your help is greatly appreciated as we work to improve the care we provide for our patients at the Stronach Regional Cancer Centre.

1.      I learned helpful tips about

a.      Healthy eating.

1                                     2                               3                                  4                               5

Strongly Disagree                   Neither Agree/ Disagree                                Strongly Agree

b.      Exercise.

1                                     2                               3                                  4                               5

Strongly Disagree                   Neither Agree/ Disagree                                Strongly Agree

c.       Stress Relief.

1                                     2                               3                                  4                               5

Strongly Disagree                   Neither Agree/ Disagree                                Strongly Agree

d.      Community Resources.

1                                     2                               3                                  4                               5

Strongly Disagree                   Neither Agree/ Disagree                                Strongly Agree

2.      The information was applicable to me.

1                                     2                               3                                  4                               5

Strongly Disagree                   Neither Agree/ Disagree                                Strongly Agree

3.      I am more confident in myself since completing this educational activity.

1                                     2                               3                                  4                               5

Strongly Disagree                   Neither Agree/ Disagree                                Strongly Agree

4.      Commitment to change (select one):

a.       I will make changes as a result of this educational activity.

b.      I am considering a change.

c.       This educational activity confirms what I am already doing.

d.      I am not yet convinced that any change is needed.

e.       I feel there may be barriers for making changes.

5.      What information from this educational activity do you plan to use?

___________________________________________________________________

6.         What barriers are there to making changes?

___________________________________________________________________

7.         This was an effective method to learn.

1                                     2                               3                                  4                               5

Strongly Disagree                   Neither Agree/ Disagree                                Strongly Agree

8.         Please list what was most useful regarding the survivorship session.

___________________________________________________________________

9.         Please list any suggestions for improving the survivorship session. 

___________________________________________________________________

10.     Is there any more information that you would have liked to know about/ any additional comments.

___________________________________________________________________

Appendix C

Survivorship Post-Radiation Therapy: Staff Evaluation Form

Please help us to improve this survivorship session by completing this evaluation form. Your help is greatly appreciated as we work to improve the care we provide for our patients at the Stronach Regional Cancer Centre.

1.      I was able to complete the survivorship sessions in a timely manner.

1                                     2                               3                                  4                               5

Strongly Disagree                   Neither Agree/ Disagree                                Strongly Agree

2. The survivorship sessions were easily incorporated within my schedule.

1                                     2                               3                                  4                               5

Strongly Disagree                   Neither Agree/ Disagree                                Strongly Agree

3.  I feel as though the sessions were useful to the patients

1                                     2                               3                                  4                               5

Strongly Disagree                   Neither Agree/ Disagree                                Strongly Agree

4. I believe that there is value in continuing on with these sessions in the future.

1                                     2                               3                                  4                               5

Strongly Disagree                   Neither Agree/ Disagree                                Strongly Agree

5. My workflow in PRPA was not negatively impacted by the sessions.

1                                     2                               3                                  4                               5

Strongly Disagree                   Neither Agree/ Disagree                                Strongly Agree

1.      What worked well in the trial of the survivorship session.

___________________________________________________________________

2.      Please list any suggestions for improving the survivorship session or barriers in moving forward. 

___________________________________________________________________

3.      Do you see the survivorship sessions being feasible going forward on a larger scale (ie: all breast patients/all patients)?

___________________________________________________________________
